# Investigation of the Potential of Repurposing Medium-Density Fiberboard Waste as an Adsorbent for Heavy Metal Ion Removal

**DOI:** 10.3390/ma17143405

**Published:** 2024-07-10

**Authors:** Kavitha H. Ranaweera, Megan N. C. Grainger, Amanda French, Narayana Sirimuthu, Michael Mucalo

**Affiliations:** 1 School of Science, University of Waikato, Hamilton 3240, New Zealand; kr140@students.waikato.ac.nz (K.H.R.); megan.grainger@waikato.ac.nz (M.N.C.G.); 2 Pacific Northwest National Laboratory, Richland, WA 99354, USA; amanda.french@pnnl.gov; 3 Department of Chemistry, Faculty of Applied Sciences, University of Sri Jayewardenepura, Nugegoda 10250, Sri Lanka; nmssirimuthu@sjp.ac.lk

**Keywords:** adsorption, heavy metals, MDF waste, regeneration, single and multi-metal ion systems

## Abstract

Medium-density fiberboard (MDF) waste generation has increased steadily over the past decades, and therefore, the investigation of novel methods to recycle this waste is very important. The potential of repurposing MDF waste as an adsorbent for the treatment of Cd(II), Cu(II), Pb(II), and Zn(II) ions in water was investigated using MDF offcuts. The highest adsorption potential in single-metal ion solution systems was observed for Pb(II) ions. The experimental data of Pb(II) ions fit well with the Freundlich isotherm and pseudo-second-order kinetic models. Complexation and electrostatic interactions were identified as the adsorption mechanisms. The adsorption behavior of multi-metal ion adsorption systems was investigated by introducing Cd(II) ions as a competitive metal ion. The presence of the Cd(II) ions reduced the adsorption potential of Pb(II) ions, yet the preference for the Pb(II) ions remained. Regeneration studies were performed by using 0.1 M HCl as a regeneration agent for both systems. Even though a significant amount of adsorbed metal ions were recovered, the adsorption potential of the MDF was reduced in the subsequent adsorption cycles. Based on these results, MDF fines have the potential to be used as an economical adsorbent for remediation of wastewater containing heavy metal ions.

## 1. Introduction

Medium-density fiberboard (MDF) is an engineered wood product made of wood fiber mixed with resin and wax that is hot-pressed into a panel shape [1]. MDF is commonly used in construction and furniture manufacturing due to its cost-effectiveness and ease of customization. As a result, global production of MDF has increased steadily over the years. For instance, MDF production was about 8 million m^3^ in 1995, which grew to almost 100 million m^3^ in 2018 [1]. Consequently, a large amount of MDF waste, such as offcuts, sawdust, and discarded MDF panels, is generated through manufacturing, construction, and demolition processes and most likely goes to landfills. For instance, the literature reports that about 25% of MDF production turns into waste in different ways, such as offcuts, damages during transportation and storage, and machining errors, in a year. After 45 years, 99% of the MDF produced currently can be considered as waste [2]. Disposal of MDF waste in landfills is a waste of energy and materials. Also, these waste materials can contain substances that can be harmful to the environment [3].

The most sustainable solution to address this problem is recycling MDF waste, as it helps to reduce the strain on landfills and lowers the demand for new raw materials, contributing to a more sustainable approach to waste management. According to a review by Zimmer and Bachmann, MDF recycling technologies can be grouped into three categories: physically recycling the materials, converting waste to energy, and chemically recycling the materials [3]. The physical recycling process involves disintegrating panels to expose the fibers by grinding and carrying out treatments for further separation of the constituents [3]. For instance, Roffael et al. investigated the effect on the mechanical and physical properties of MDF when virgin fibers were replaced with recycled fibers. They observed that MDF produced from 100% recycled fibers had considerably lower thickness swelling values and lower formaldehyde release [4]. MDF waste has also been used for composite production. Kreutz et al. obtained a composite by incorporating MDF waste in virgin polystyrene. Their results indicated that the degradation was slowed down by the presence of MDF residue [5]. Also, MDF waste can be used for the production of energy (heat and electricity) via a combustion process [6]. However, this should occur in controlled facilities to reduce toxic gas emissions. The (bio)chemical recycling process involves breaking down the components in the material to promote transformations in the chemical structure of the material. For instance, Zazycki et al. produced char from MDF waste and evaluated its ability to remove Food Red 17 (FR17) dye from effluents. The adsorbent removed 90% of FR17 at pH 2 and was efficient for eight adsorption–desorption cycles [7]. Gomes et al. investigated the adsorption capacity of activated carbon prepared from MDF via both physical activation (using CO_2_) and chemical activation (using K_2_CO_3_) towards methylene blue. The physically and chemically activated MDF showed adsorption capacities of 1.50 and 2.77 mg g^−1^, respectively [8].

Water contamination due to heavy metal ions is another serious problem faced by the modern world. Heavy metal contamination can be of both anthropogenic and natural origins. Anthropogenic origins include activities such as industrial wastewater discharge, mining, and processing, while natural origins include volcanic eruptions, soil weathering, and erosion of sediments [9,10]. These contaminations have been reported throughout the world and pose a serious threat to human health [11,12,13,14]. Previous studies have identified associations between various heavy metals and diseases. For instance, exposure to lead and cadmium has been shown to be associated with cancer and cardiovascular diseases [15,16,17]. In addition, exposure to nickel has been reported to increase mortality rates and cancer [18,19]. Exposure to high arsenic concentrations can cause cancer and toxic effects on the cardiovascular system, kidney, liver, skin, and lungs [20]. Long-term exposure to high copper concentrations can cause liver and kidney damage [21]. These findings highlight the importance of heavy metal remediation. The most common procedures available for heavy metal ion treatment include adsorption, chemical precipitation, ultrafiltration, and reverse osmosis [22]. Among these methods, adsorption is considered to be one of the best technologies due to its cost-effectiveness, high efficiency, and simple operation [23].

The development of novel, low-cost adsorbents for heavy metal ion treatment has become an emerging research field. However, most of these adsorbents have been derived from materials such as biosorbents and natural and industrial geomaterials [24]. For instance, different forms of biosorbents and geomaterials such as sawdust, eggshell, sugarcane bagasse [25], banana and orange peel [26], kaolinite clay [27], and zeolite [28] have been investigated in previous studies for heavy metal ion treatment. However, the potential of repurposing construction and demolition waste (CDW) materials as adsorbents for heavy metal ion treatment has not been fully investigated yet [24]. Recycling of CDW as adsorbents is a winning strategy as it helps in both waste recycling and environmental remediation. These research studies are also beneficial for attaining circular economy goals.

In this study, we aim to evaluate the potential of repurposing MDF waste (MDF offcuts) as a low-cost material for heavy metal ion remediation in an attempt to introduce a new adsorbent for heavy metal ion treatment and a new recycling method for MDF waste. The objectives of this project were to: (1) compare the adsorption potential of MDF fines towards Cd(II), Cu(II), Pb(II), and Zn(II) ions; (2) model the adsorption process; (3) explore the adsorption mechanisms; and (4) investigate the regeneration potential of MDF fines. To the best of our knowledge, the adsorption capacity of MDF fines towards these metal ions has not been studied in great depth, and hence, this represents seminal research in this area.

## 2. Materials and Methods

### 2.1. Preparation of MDf Fines

MDF offcuts were provided by request from Hexion NZ Limited (Mount Maunganui, New Zealand). The collected offcuts were crushed using a hammer to obtain smaller pieces in order to facilitate the grinding process. The obtained materials were ground using a grinder (Bosch) and sieved (Matest, mesh sizes: 0.425 mm and 1 mm) to collect a fraction of 0.425–1 mm. This particle size range was selected for the study as, in practical applications, powder-like materials (very low particle size) cause higher pressure drops in filter beds. The separated fraction was used in the adsorption experiments without any chemical or physical modifications. The prepared materials were stored in sealed plastic bags for further experiments.

### 2.2. Characterization of MDF Fines

The surface morphology of MDF fines and elemental composition prior to and after treatment were evaluated using a Hitachi Regulus SU8230 Field Emission Scanning Electron Microscope (Hitachi High-Tech, Ibaraki, Japan). The samples were coated with platinum to minimize charging whilst under the beam. The distribution of the functional groups on the MDF fines’ surface was evaluated using a PerkinElmer Spectrum 400 Fourier transform infrared (FTIR) spectrometer (PerkinElmer, Shelton, CT, USA) using the KBr disk method (scanning range: 4000–450 cm^−1^ and resolution: 4 cm^−1^). The surface area of the MDF fines was estimated using an Autosorb iQ (Quantachrome, Boynton Beach, FL, USA) surface area analyzer. Prior to the analysis, MDF fines were degassed at 105 °C for 18 h to remove residual volatiles and moisture. The isotherm data were collected using N_2_ gas in the relative pressure range of 10^−3^–1. The X-ray diffractogram was recorded on a Panalytical Empyrean multi-purpose X-ray diffractometer in the scanning range of 5°–70° 2θ (Panalytical, Malvern, UK). Thermogravimetric analysis of the sample was carried out using an SDT 650 thermal analyzer (TA instruments, New Castle, DE, USA) in the temperature range of 30–800 °C at a 10 °C min^−1^ heating rate.

### 2.3. Preparation of Stock Solutions

Cd(II), Cu(II), Pb(II), and Zn(II) ions stock solutions of 500 mg L^−1^ were prepared using Cd(NO_3_)_2_, 4H_2_O (BDH chemicals, Poole, England), Cu(NO_3_)_2_.2.5H_2_O (Ajax Finechem, NSW, Australia), Pb(NO_3_)_2_ (Ajax Finechem, NSW, Australia), and Zn(NO_3_)_2_.6H_2_O (Thermo Scientific, Fair Lawn, NJ, USA), respectively. Analytical-grade chemicals and reagents were used for the study, and type 1 water (distilled and deionized; 18.2 MΩ cm resistivity) was used for the dilution of metal ion solutions. The initial adsorbate concentrations in the solutions were determined using Agilent Technologies 8900 Inductively Coupled Plasma Mass Spectrometry (ICP-MS) (Agilent Technologies, Santa Clara, CA, USA) to confirm their values before conducting adsorption experiments.

### 2.4. Batch Adsorption Analysis

The adsorption capacity of MDF fines to the targeted metal ions (Cd(II), Cu(II), Pb(II), and Zn(II) ions) was evaluated separately in single-metal ion solution systems. These experiments were carried out by mixing 50 mL of each metal ion solution, with a 25 mg L^−1^ initial metal ion concentration (pH = 5.5), with 0.25 g of MDF fines in 125 mL reagent bottles. The samples were shaken in an orbital shaker (FinePCR SH30, Seoul, Republic of Korea) at 120 rpm for 4 h at room temperature (20 ± 3 °C). The resulting metal ion solution was separated by gravity filtration. The separated supernatants were diluted to achieve measurable concentration ranges and filtered using 0.45 µm cellulose acetate syringe filters. The samples were acidified (2%) using nitric acid to prevent metal ion precipitation, and the metal ion concentration was analyzed by ICP-MS.

The Pb(II) ion adsorption kinetics on MDF fines were evaluated by adding 50 mL of 25 mg L^−1^ Pb(II) ion solution (pH = 5.5) and 0.25 g of MDF fines to a reagent bottle. The mixtures were shaken at 120 rpm at room temperature and removed after predetermined time periods (5, 15, 30, 60, 120, and 240 min). The remaining metal ion concentrations were determined via ICP-MS analysis using the same method as above. The adsorption kinetics data were fitted to pseudo-first-order, pseudo-second-order, and Elovich kinetic models. The effect of competing metal ions (multi-metal ion solution systems) was analyzed by mixing 25 mL of 50 mg L^−1^ Pb(II) ion solution with 0.25 g of MDF fines at pH 5.5. Then, 25 mL of 50 mg L^−1^ Cd(II) ion solution was added to the adsorption system. The adsorption kinetics were analyzed following the same procedure.

The Pb(II) ion adsorption isothermal studies were carried out by preparing Pb(II) ion solutions of different concentrations (25, 50, 100, 250, and 200 mg L^−1^) at pH 5.5, and each solution (50 mL) was added to 0.25 g of MDF fines. The samples were shaken (120 rpm) in an orbital shaker at room temperature for 4 h. The residual Pb(II) ion concentrations in the supernatants were determined by ICP-MS analysis. The adsorption isotherms were fitted using Freundlich, Langmuir, and Temkin isotherm models.

The effect of the pH on the adsorption of Pb(II) ions was studied by mixing 50 mL of the 25 mg L^−1^ metal ion solutions with 0.25 g MDF fines. The pH values of the solutions were adjusted to 3.3, 4, 4.5, 5, 5.5, and 6 using 0.1 M NaOH and HCl solutions. The study was limited to pH values ≤6 as Pb(II) ions precipitate out of solution, forming less soluble hydroxyl complexes, such as Pb(OH)_2_, at higher pH values [29]. The effect of the dose of MDF fines was evaluated by mixing 50 mL of 25 mg L^−1^ metal ion solutions with different doses of MDF fines (0.10, 0.15, 0.2, 0.25, 0.3 g) at pH 5.5. The mixtures were shaken at 120 rpm at room temperature, and the Pb(II) ion concentrations in the supernatants were determined by ICP-MS analysis.

For desorption experiments, the previously Pb(II) ion-loaded adsorbent was washed with type 1 water to remove any unadsorbed metal ions. The samples were oven-dried (Contherm series 2000. Contherm Scientific, Wellington, New Zealand) at 40 °C for 24 h, after which 0.25 g of adsorbent was added to 20 mL of 0.1 M HCl solution and shaken at 120 rpm for 4 h. The concentration of the metal ions released by desorption from the adsorbent was analyzed by ICP-MS.

The experimental conditions for these experiments were selected based on the data reported in the literature [30,31,32]. Blank analyses were also conducted for each experiment to measure whether any adsorption of metal ions occurred on the walls of the glassware and during the filtration process. This analysis was performed by agitating all the reagents used in the experiment except the adsorbent. After completion of the required time for the experiment, the residual metal ion concentration was analyzed using ICP-MS.

An experiment was also carried out to evaluate whether MDF fines leached out potentially harmful inorganic or organic substances into the treated water. This analysis was performed by agitating 0.25 g of the adsorbent for 4 h with 50 mL of type 1 water in an orbital shaker at 120 rpm. The concentrations of Al, Ba, Ca, Cd, Co, Cr, Cu, Fe, Hg, K, Mg, Mn, Na, Ni, P, Pb, Sr, U, V, and Zn in the treated water were analyzed using ICP-MS. The total organic carbon (TOC) of the treated water was additionally measured using the chemical oxidation method in an Aurora 1030 TOC wet oxidation analyzer (OI Analytical, College Station, TX, USA). All of the aforementioned experiments were performed in triplicate.

### 2.5. Calculations and Data Analysis

The % removal and adsorption capacity values were calculated using Equations (1) and (2), respectively [32].

(1) $$\%removal=\left[\frac{C_{o}-C_{t}}{C_{o}}\right]\times 100$$

(2) $$q_{e}=\frac{\left(C_{0}-C_{e}\right)V}{W}$$

where *C*_0_ and *C_t_* are the initial and final adsorbate concentration (mg L^−1^), $q_{e}$ is the adsorption capacity of MDF fines at equilibrium (mg g^−1^), *C_e_* is the adsorbate concentration at equilibrium (mg L^−1^), *V* is the adsorbate volume (L), and *W* is the mass of the MDF fines (g).

Adsorption kinetics studies were carried out by fitting the experimental data to the pseudo-first-order [33], pseudo-second-order [34], and Elovich [35] kinetic model equations, which are expressed by Equations (3), (4), and (5), respectively.

(3) $$q_{t}=q_{e}(1-e^{-k_{1}t})$$

(4) $$q_{t}=\frac{k_{2}q_{e}^{2}t}{1+k_{2}q_{e}t}$$

(5) $$q_{t}=\frac{1}{\beta }\ln\left(1+\alpha \beta t\right)$$

where *k*_1_ (min^−1^) refers to the equilibrium rate constant of pseudo-first-order adsorption, *q_t_* is the adsorption capacity at time *t* (mg g^−1^), *k*_2_ is the rate constant for pseudo-second-order adsorption (g mg^−1^ min^−1^), α is the initial sorption rate in the Elovich model (mg g^−1^ min^−1^), and β is a constant related to the extent of surface coverage and the activation energy for chemisorption in the Elovich model (g mg^−1^).

Adsorption isothermal studies were carried out by fitting the experimental data to the Langmuir [36], Freundlich [37], and Temkin [38] isotherm models, which are represented by Equations (6), (7), and (8), respectively.

(6) $$q_{e}=\frac{q_{m}bC_{e}}{1+bC_{e}}$$

(7) $$q_{e}=KC_{e}^{1/n}$$

(8) $$q_{e}=\frac{RT}{B}\ln K_{T}C_{e}$$

where *q_m_* represents the maximum adsorption capacity obtained from the Langmuir isotherm model (mg g^−1^); *C_e_* denotes the equilibrium metal ion concentration (mg L^−1^); *b* and *K* are the Langmuir equilibrium constant (L mg^−1^) and Freundlich isotherm constant, respectively; 1/*n* is the heterogeneity factor (related to the adsorption intensity); *R* is the universal gas constant (J K^−1^ mol^−1^); *T* is the temperature (K); *B* is a constant related to the sorption heat (kJ mol^−1^); and $K_{T}$ is the Temkin isotherm constant (L g^−1^).

The best-fit model for the experimental data was identified using R^2^ values and error functions. Closeness of the R^2^ value to unity and smaller error functions values indicated the best-fit model. The sum of squares of the error (SSE) and the hybrid fractional error function (HYBRID), which are represented by Equations (9) and (10), respectively, were used in this study [39].

(9) $$SSE=\sum_{i=1}^{n}(q_{e,cal}{-q_{e,exp})}_{i}^{2}$$

(10) $$HYBRID=\frac{100}{n-p}\sum_{i=1}^{n}{\left[\frac{{\left(q_{e,exp}-q_{e,cal}\right)}^{2}}{q_{e,exp}}\right]}_{i}$$

where *q*_*e*,*cal*_ is adsorption capacity calculated from the models (mg g^−1^), *q*_*e*,*exp*_ is the adsorption capacity found from the experiment (mg g^−1^), *p* is the number of parameters in the models, and *n* is the number of experimental data points.

All the measurements are expressed as an average of triplicate samples, and the standard deviation of the results is represented by error bars. The results are given as mean ± standard deviation. Microsoft Excel (2019) and origin Pro 9 software were used in this study.

## 3. Results and Discussion

This study aimed to evaluate the adsorption potential of MDF fines without any major modifications being involved as an effort to reduce the adsorbent preparation cost and minimize energy inputs.

### 3.1. Characterization of MDF Fines

#### 3.1.1. Fourier Transform Infrared (FTIR) Analysis

**Figure 1:**
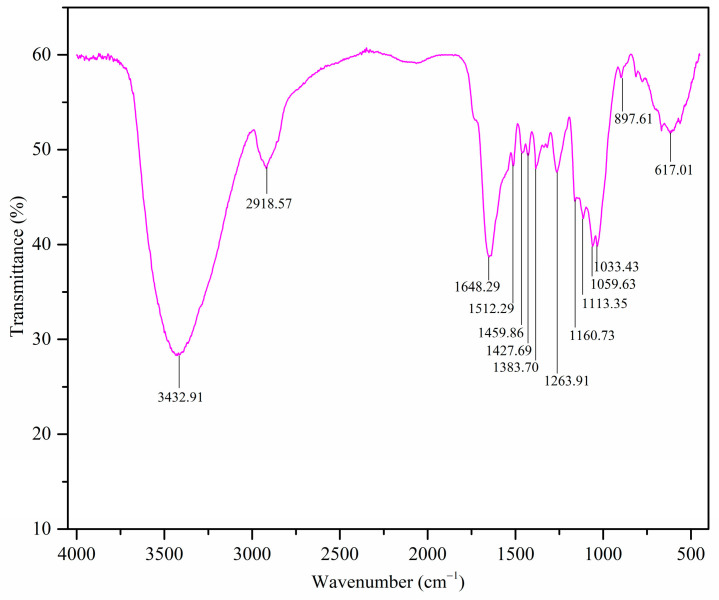
FTIR spectrum of the MDF fines used in this study.

The FTIR spectrum of the MDF fines is shown in Figure 1, and the band assignments are as summarized in Table 1.

The presence of these functional groups is important for the binding of metal ions via adsorption mechanisms such as chemisorption and physisorption. The adsorption mechanisms of MDF fines are discussed under Section 3.2.6. These functional groups are present due to the presence of cellulose, hemicellulose, and lignin in the sample, as indicated by other characterization techniques employed in this study (see Section 3.1.3 and Section 3.1.5).

#### 3.1.2. Scanning Electron Microscopy and Energy Dispersive X-ray Spectroscopy (SEM-EDX) Analysis

**Figure 2:**
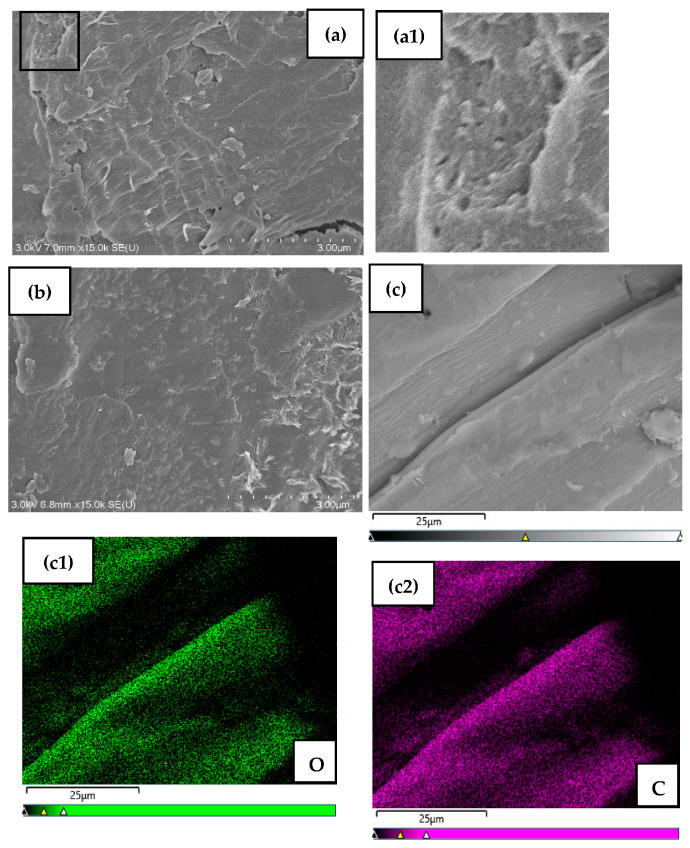

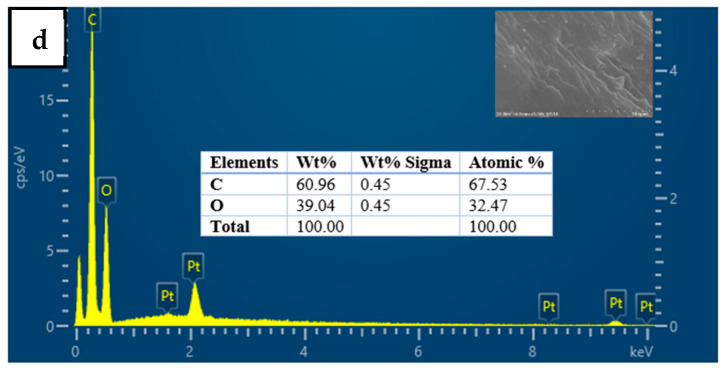
SEM micrographs (**a**,**b**), magnified image of marked area of micrograph (**a**) to show the porous nature (**a1**), SEM mapping of elemental distribution ((**c**), (**c1**)—oxygen, (**c2**)—carbon), and the EDX spectrum (**d**) of MDF fines.

The scanning electron micrographs and the energy-dispersive X-ray (EDX) spectrum of MDF fines are presented in Figure 2. The morphology of MDF fines has the appearance of a rough, irregular, and porous surface, which leads to surface area enhancement. This type of surface structure favors the adsorption process, as it provides more surface area for the binding of metal ions. The elemental analysis showed carbon and oxygen as predominant elements at 60.96% and 39.04%, respectively. Pt was detected as an element in the EDX spectrum as it was used to coat the samples for scanning electron microscopy (SEM) analysis.

#### 3.1.3. Thermogravimetric Analysis (TGA)

**Figure 3:**
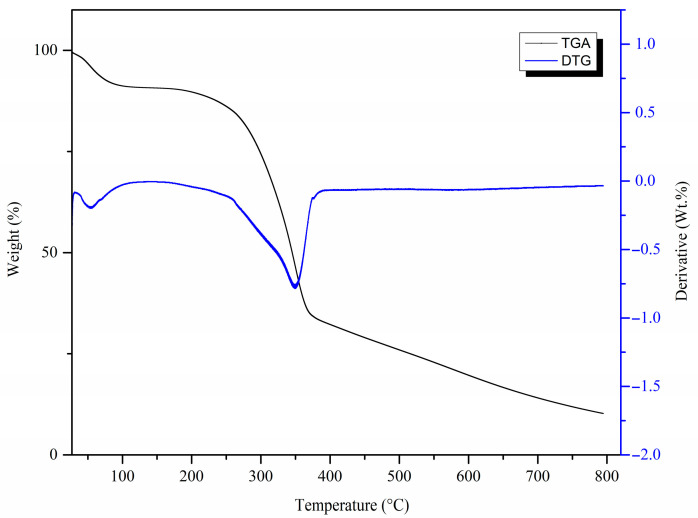
TGA and corresponding DTG curve of MDF fines.

Figure 3 shows the thermogram and corresponding derivative thermogravimetric (DTG) curve of the MDF fines. The thermogram shows two variations corresponding to two weight loss events. The weight loss of about 10% observed around 80 °C can be attributed to the loss of moisture in the sample. Significant degradation of the sample was observed around 350 °C, causing weight loss of about 60%, which was attributed to the decomposition of cellulose, hemicellulose, and lignin [42]. Similar results have been reported for other cellulose-based materials such as sawdust [43].

#### 3.1.4. Surface Area Analysis

**Figure 4:**
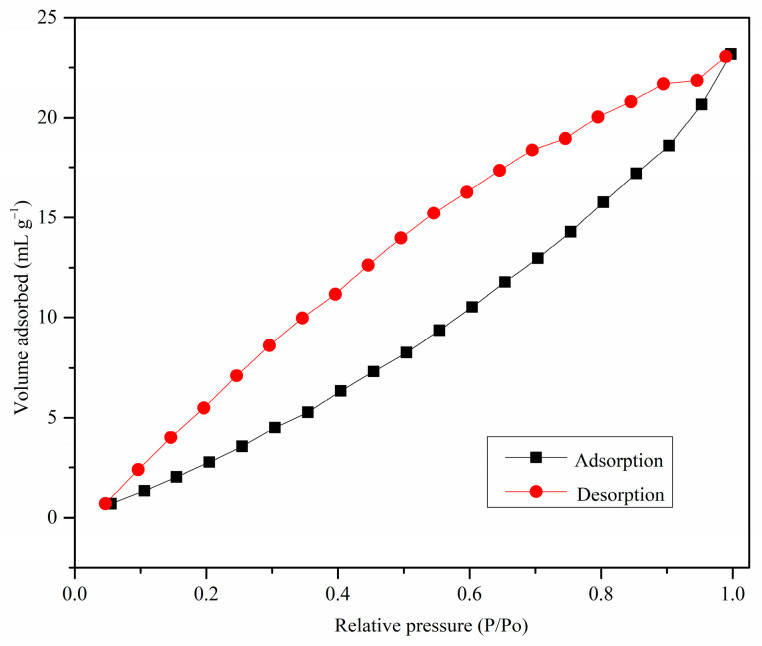
The N_2_ adsorption–desorption isotherm of MDF fines.

The N_2_ adsorption–desorption isotherm of MDF fines is shown in Figure 4. The low adsorption at low relative pressures and the presence of hysteresis indicate that this isotherm has characteristics of a type V isotherm based on the IUPAC classification. These results thus indicate that MDF is mainly a mesoporous adsorbent [44]. Low adsorption at low relative pressures were also observed for activated carbon prepared from guava seeds (carbonization temperature: 500 °C) according to the N_2_ adsorption–desorption isotherm, which showed characteristics of type III isotherm [45]. The porous nature of the adsorbent was observed under SEM analysis as well (see Section 3.1.2). Furthermore, the obtained isotherm showed characteristics of type H3 hysteresis, as limiting adsorption was not observed at high relative pressures. The presence of the hysteresis loop was observed at low relative pressure ranges as well. This may have occurred due to the swelling of non-rigid pores or entrapment of adsorbate molecules in pores of about the same width as the adsorbate molecule [46]. According to the literature, this type of adsorption behavior is observed with materials having wedge-shaped pores [47].

The most common method used in the literature for surface area analysis is the Brunauer–Emmett–Teller (BET) method [48,49]. However, this method cannot be applied to a type V-shaped isotherm due to the absence of an inflection point. Therefore, the Barrett–Joyner–Halenda (BJH) method was used for the calculation of the surface area [50]. The calculated surface area, average pore radius, and total pore volume according to the BJH method were 21.9 m^2^ g^−1^, 2.3 nm, and 0.036 cm^3^ g^−1^. However, the BJH method does not include the surface area contributed by micropores, so the actual surface area could be higher than the calculated surface area [46].

#### 3.1.5. X-ray Diffraction (XRD) Analysis

**Figure 5:**
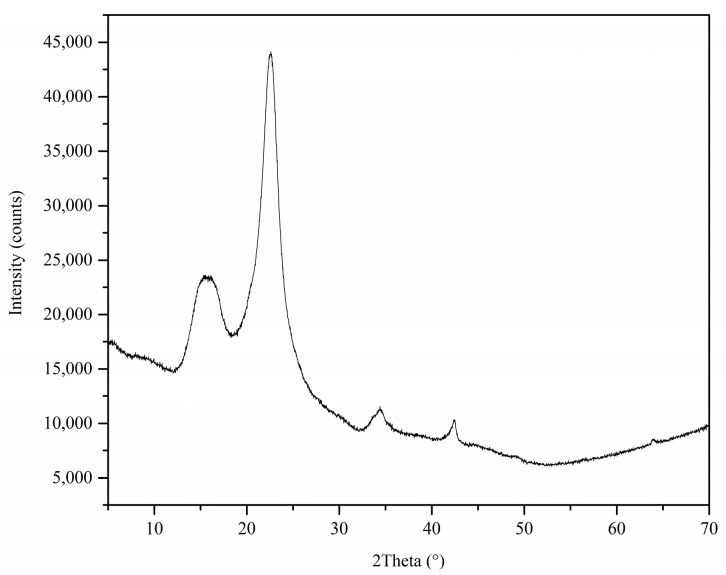
X-ray diffractogram of MDF fines.

The absence of sharp peaks in the X-ray diffractogram of the MDF fines (Figure 5) indicates the poorly crystalline nature of the cellulose-based adsorbent. The high-intensity peaks observed around 2 theta values of 16° and 23° indicate the presence of cellulose in the sample. Similar observations have been reported in a previous study which analyzed the X-ray diffractogram of durian wood sawdust. That study also observed sharp and narrow peaks around 2 theta values of 16° and 22°, which were attributed to the presence of cellulose in the sample [51]. The low-intensity peaks observed between 2 theta values of 30°–70° may have occurred due to the resin material present in the sample. Similar observations were reported in a study that analyzed the X-ray diffractogram of urea formaldehyde [52].

Based on the characterization analysis, MDF is a porous material with an irregular surface structure. Cellulose is the main component, and other constituents may include hemicellulose, lignin, and resin materials.

### 3.2. Adsorption Experiments

Blank analysis confirmed that metal ions do not become adsorbed onto the walls of glassware during the filtration process. The metal ion concentration in the treated water was tested as explained in Section 2.4. All the tested metal ion concentrations in the treated water were much lower than WHO drinking water standards based on the results. However, the TOC concentration was 90.3 mg L^−1^ in the treated water. This may have occurred due to the leaching of organic and resin matter during the adsorption process [53]. This is a disadvantage for the adsorption process. If this material is used for industrial-scale applications, the TOC of the treated water must be monitored. The literature reports that the maximum permissible limits for TOC for wastewater discharging into natural water bodies and for irrigation are 200 and 250 mg L^−1^, respectively, according to Indian standards [54].

#### 3.2.1. Effect of Metal Ion Type

**Figure 6:**
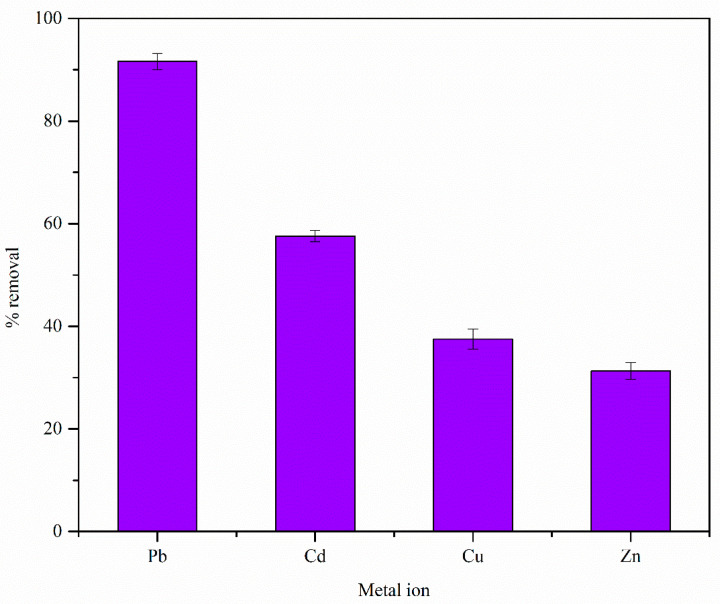
Effect of metal ion type on the adsorption process on MDF fines in single-metal ion solution systems (contact time: 4 h, adsorbent dose: 0.25 g, pH: 5.5, shaking speed: 120 rpm, initial metal ion concentration: 25 mg L^−1^).

Figure 6 shows the effect of the metal ion type on the adsorption process. The adsorption performance followed the order of Pb(II) > Cd(II) > Cu(II) > Zn(II). The literature reports that the adsorption performance of an adsorbate can be controlled by several factors, such as electronegativity and the hydrated ionic radius of the adsorbent [30,55,56]. These physiochemical properties of the analyzed metal ions are given in Table 2 [57].

Previous studies have reported that metal ions with high electronegativity easily interact with the negatively charged binding sites on the adsorbent surface, yielding high adsorption capacity values [56]. Metal ions with larger hydrated radii have more difficulty accessing adsorption sites and forming bonds with the functional groups. Therefore, metal ions with a smaller hydrated radius are expected to adsorb more readily compared to metal ions with a larger hydrated radius [30,55]. These factors help to explain the adsorption sequence of Pb(II) > Cu(II) > Zn(II). For instance, Pb(II) ions have the highest electronegativity (2.33) among the studied metal ions (see Table 2). Therefore, Pb(II) ions can interact with the functional groups present on MDF fines (see Figure 11) more easily than the other metal ions. As Pb(II) ions have a smaller hydrated radius compared to other metal ions, Pb(II) ions can transport through the pore network of MDF fines and access binding sites that are not accessible to other metal ions. Preferential adsorption of Pb(II) ions has been observed for other adsorbents, such as xanthate-modified corn cob, xanthate-modified chestnut shell [58], and cellulose and lignin extracted from rice bran [59]. However, MDF fines showed a higher adsorption potential towards Cd(II) ions than Cu(II) ions, and the adsorption behavior for these ions cannot be explained by comparing electronegativity and hydrated radius values (see Table 2). The literature reports that other cellulose-based or charred cellulose-based adsorbents, such as chemically modified olive stone activated carbon [55], cabbage waste [60], and corncob cellulose [61], also exhibit higher adsorption potential for Cd(II) ions compared to Cu(II) ions.

The results of this study suggest that the adsorption behavior of MDF fines cannot be explained solely based on the physiochemical properties of the adsorbate. The properties of the adsorbent, such as the types of functional groups present on the adsorbent surface and distribution of the functional groups, may have also affected the adsorption behavior. Therefore, further studies will be required in order to explain this adsorption behavior. As the MDF fines showed higher adsorption capacity towards Pb(II) ions, Pb was selected as the main metal ion for involvement in the other adsorption studies.

#### 3.2.2. Adsorption Kinetics—Single- and Multi-Metal Ion Systems

##### Single-Metal Ion Systems

**Figure 7:**
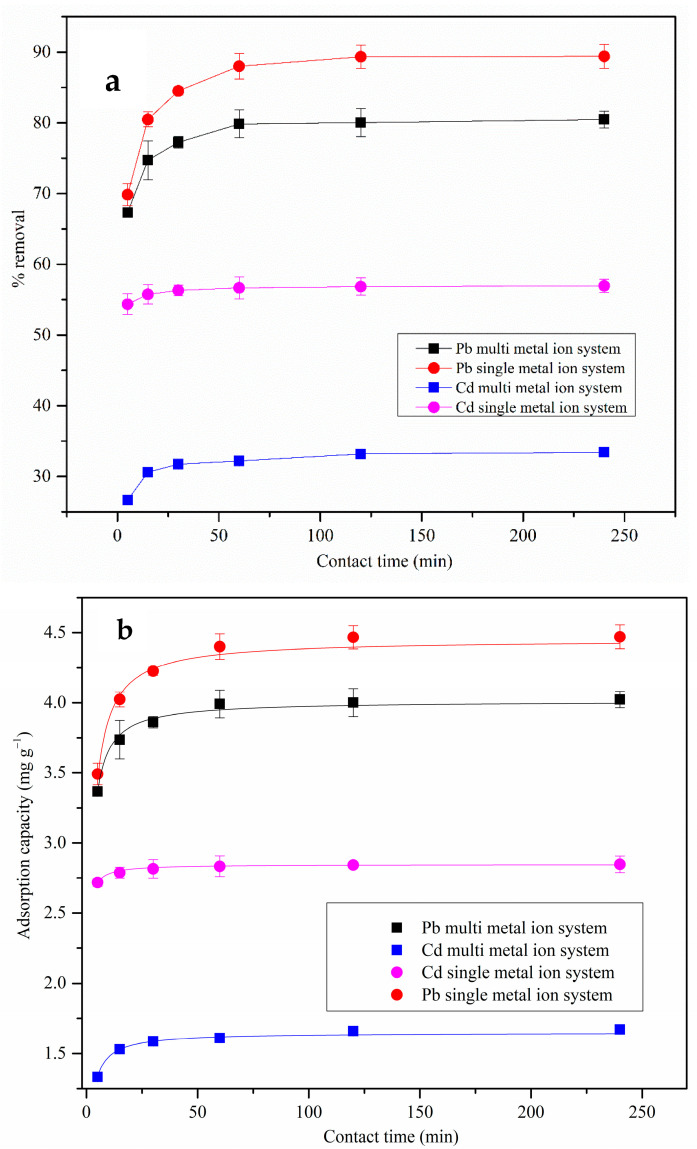
(**a**) Effect of contact time on Pb(II) and Cd(II) ion removal in single- and multi-metal ion solution systems by MDF fines (pH: 5.5, adsorbent dose: 0.25 g, initial metal ion concentration: 25 mg L^−1^, shaking speed: 120 rpm). (**b**) The pseudo-second-order kinetic model fitting to Pb(II) and Cd(II) ion adsorption onto MDF fines in single- and multi-metal ion solution systems.

Kinetic modeling of metal ion adsorption onto MDF fines was performed by fitting the experimentally obtained effect of contact time data to the non-linear pseudo-first-order, pseudo-second-order, and Elovich kinetic models. Based on the effect of contact time data, the MDF fines exhibited a rapid removal of Pb(II) ions, which may have occurred due to the large number of functional groups present on the surface at the initial stages (see Figure 7a). The adsorption system reached a plateau after 60 min, reaching an equilibrium. This indicates that the binding sites on the MDF fines surface were saturated. A maximum removal of 89 ± 1.7% was recorded for Pb(II) ion removal at equilibrium for a 25 mg L^−1^ initial metal ion concentration. The kinetic fitting results are presented in Table 3 and Figure 7b. The Pb(II) ion adsorption behavior onto MDF fines was explained best by the pseudo-second-order kinetic model, indicating that the rate-limiting step on the MDF fines was chemisorption of the metal ions.

##### Multi-Metal Ion Systems

The presence of competitive metal ions can affect the adsorption behavior. In this study, this effect was analyzed using multi-metal ion systems containing Pb(II) and Cd(II) ions (25 mg L^−1^). Figure 7a presents the % removal of Pb(II) and Cd(II) ions in single- and multi-metal ion adsorption systems. The Pb(II) ion removal was higher than that for Cd(II) in this multi-metal ion system, which was also observed in the single-metal ion systems (see Section 3.2.1). The high electronegativity and smaller hydrated ionic radius may have led to better adsorption of Pb(II) ions compared to the Cd(II) ions (see Table 2). However, as discussed in Section 3.2.1, adsorption mechanisms can be complex, and other factors, such as the nature and the distribution of the functional groups on the adsorbent surface, may have also affected the preferential adsorption of Pb(II) ions. Based on these results, the % removal values of both metal ions decreased in the multi-metal ion system compared to the single-metal ion systems due to the competition for the binding sites. However, the reduction in the % removal was significantly higher for Cd(II) ions compared to the Pb(II) ions.

The pseudo-second-order kinetic model fit well with the experimental data of multi-metal ion systems, as shown in Figure 7b and Table 3. These results indicate that chemisorption is the rate-limiting step for these adsorption systems.

#### 3.2.3. Adsorption Isothermal Studies

**Figure 8:**
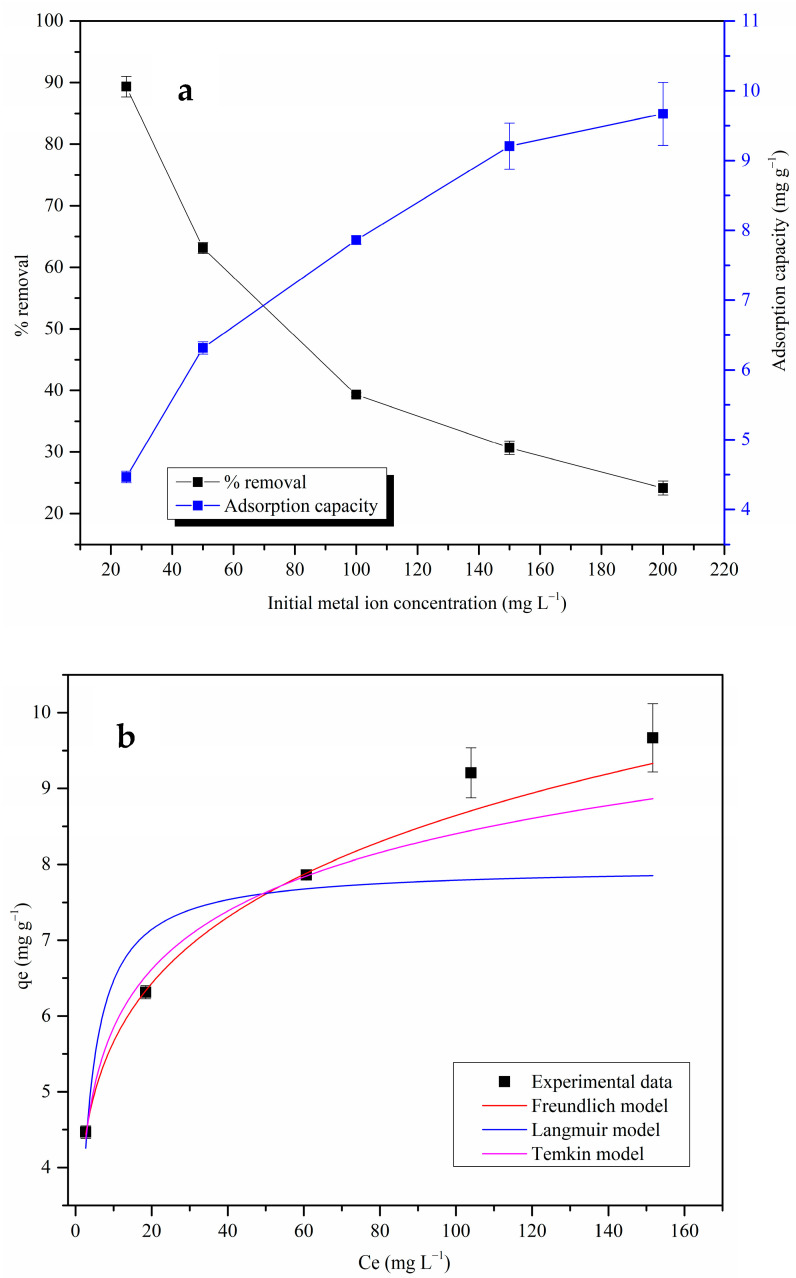
(**a**) Effect of initial metal ion concentration on Pb(II) ion removal by MDF fines (pH: 5.5, adsorbent dose: 0.25 g, contact time: 4 h, shaking speed: 120 rpm); (**b**) adsorption isothermal models of Pb(II) ion adsorption onto MDF fines.

The % removal values decreased and the adsorption capacity values increased with the increase in the initial Pb(II) ion concentration, as shown in Figure 8a. The adsorption capacity values were low at low Pb(II) ion concentrations due to the barrier in the mass transfer of Pb(II) ions between the metal ion solution and the surface of the MDF fines. Higher initial Pb(II) ion concentrations facilitated the Pb(II) ion diffusion process, increasing the adsorption capacity of MDF fines [62]. The ratio of the metal ions to binding sites was high at higher initial metal ion concentrations, and the amount of free metal ions remaining in the adsorbate solution increased with an increase in the initial Pb(II) ion concentration. This led to a decrease in the % removal values in the higher initial metal ion concentrations, even though the amount of Pb(II) ions adsorbed per unit mass of the MDF fines increased (increasing adsorption capacity).

The experimental data were fitted to isotherm models as shown in Figure 8b, and the obtained data are summarized in Table 4. The better fitting of the experimental data with the Freundlich isotherm model indicates that the adsorption of Pb(II) ions takes place with multilayer formation on a heterogenous adsorbent surface [63]. If the Freundlich isotherm constant, *n*, satisfies the condition 0 < *n* < 10, then the adsorption process is described as a favorable adsorption [64]. The calculated n value was 5.44, indicating that the adsorption process is indeed favorable.

#### 3.2.4. Effect of pH

**Figure 9:**
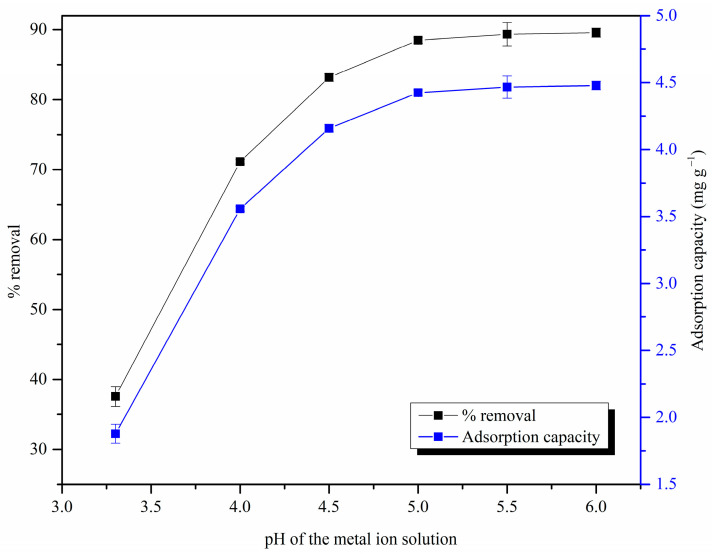
Effect of pH on Pb(II) ion removal by MDF fines (adsorbent dose: 0.25 g, initial metal ion concentration: 25 mg L^−1^, contact time: 4 h, shaking speed: 120 rpm).

As shown in Figure 9, the % removal and adsorption capacity increased when the pH was increased from 3.3 to 6. The analysis was limited to solution pH values lower than 6, as Pb(II) ions might otherwise precipitate in such media, forming less soluble complexes such as Pb(OH)_2_ [65]. The adsorption potential was significantly low at low pH values compared to the high pH values due to the protonation of active sites on the adsorbent surface (cellulose-OH_2_^+^) [66]. At higher pH values, the negative charges on the adsorbent surface increased, increasing the electrostatic effect and leading to higher adsorption capacity values. However, the adsorption capacity values did not change significantly beyond pH 5, suggesting that, even though the electrostatic interactions are an important adsorption mechanism of the MDF fines for adsorption of Pb(II) ions, they are not the main adsorption mechanism. Similar observations were reported in a previous study [32]. The maximum adsorption capacity was observed at pH 6 and was 4.48 ± 0.03 mg g^−1^.

#### 3.2.5. Effect of Adsorbent Dose

**Figure 10:**
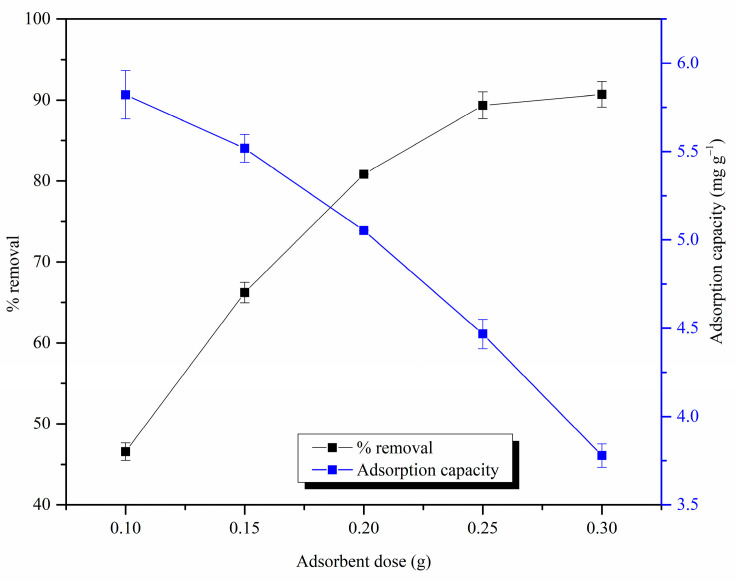
Effect of adsorbent dose on Pb(II) ion removal by MDF fines (initial metal ion concentration: 25 mg L^−1^, pH: 5.5, contact time: 4 h, shaking speed: 120 rpm).

As shown in Figure 10, the % removal values increased with the increase in the adsorbent dose and nearly stayed constant after 0.25 g. This may have occurred due to the availability of more binding sites at high adsorbent doses and, eventually, with the adsorption system reaching equilibrium. However, the adsorption capacity values decreased with the increase in the adsorbent dose. It can be observed that the improvement in % removal values gradually decreased when the adsorbent dosage increased, causing a reduction in the adsorption capacity. For instance, an increase in the adsorbent dose from 0.1 g to 0.15 g increased the % removal by 19%, whereas an increase in the adsorbent dosage from 0.2 g to 0.25 g only improved the % removal by 8%. At a constant Pb(II) ion concentration and high dosage of MDF fines, due to the presence of a large number of binding sites, Pb(II) ions were unable to occupy all sites, leading to the observed lower adsorption capacity [55].

#### 3.2.6. Adsorption Mechanisms

**Figure 11:**
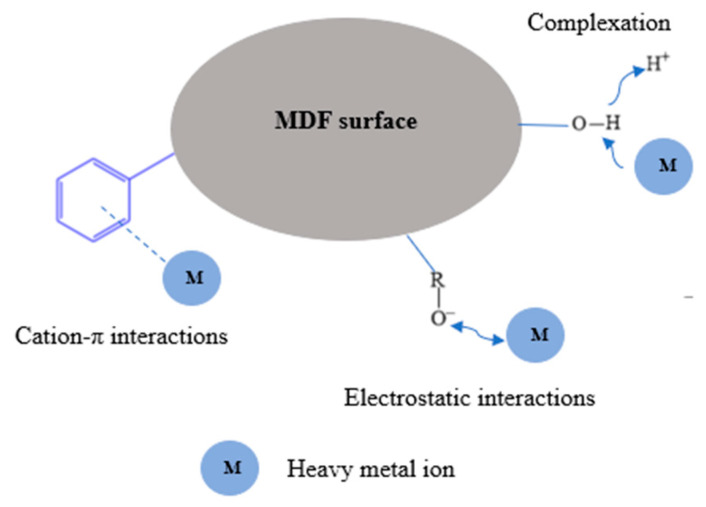
Mechanisms involved in the metal ion adsorption processes on MDF fines [32].

As shown in Figure 11, it can be suggested that heavy metal ions interact with MDF fines via adsorption mechanisms such as complexation and electrostatic interactions. These mechanisms were suggested based on the adsorbent characterization and adsorption analyses.

The fitting of experimental data to the pseudo-second-order kinetic model suggests the involvement of chemisorption mechanisms such as complexation in the adsorption process [67]. The functional groups on the MDF fines surface, which can act as electron donors, form coordination complexes with the heavy metal ions during complexation (see Figure 11). The ability of heavy metal ions to bind to the surface of MDF fines via electrostatic interactions was indicated by the presence of functional groups such as O-H, C-O, and aromatic groups. The involvement of electrostatic interactions was indicated when analyzing the effect of pH on the adsorption process (see Section 3.2.4) as well. Based on the results, it can be suggested that complexation and electrostatic interaction mechanisms are involved in the adsorption process.

**Figure 12:**
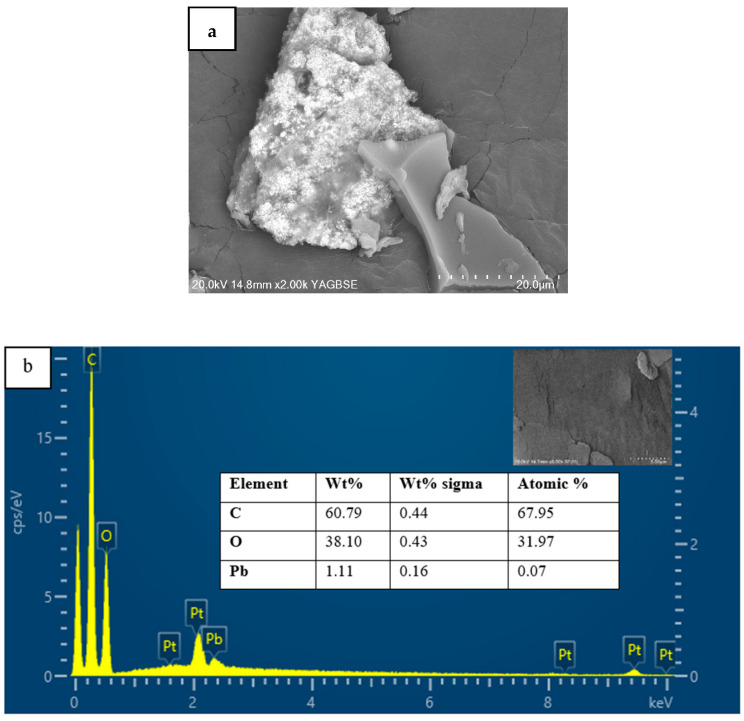
(**a**) Backscatter electron image and (**b**) EDX spectrum of Pb(II) ion-treated MDF fines.

Figure 12a shows a backscattered electron SEM image of the surface of MDF fines after the Pb(II) ion adsorption process. The Pb(II) ion-exposed adsorbent was washed with type 1 water to remove any unadsorbed excess Pb(II) ions present on the surface, then dried prior to performing the SEM analysis.

The backscattered electron image showed brighter regions, indicating the presence of heavy elements. Based on EDX analysis (Figure 12b), Pb was indicated as a constituent, demonstrating the binding of Pb(II) ions onto the adsorbent surface. The presence of Pt was merely as a consequence of coating the samples to facilitate image acquisition.

#### 3.2.7. Desorption of Metal Ions and Regeneration of MDF Fines

The ability to regenerate and reuse is very important, as it determines the economic success of an adsorbent [68]. The concentrated metal ion solutions obtained as a result of the regeneration process can be used to recover metal ions that may be able to be used in other applications [69]. According to the literature, research studies on adsorbent regeneration are very limited, and therefore, performing desorption analysis is very important [70]. In this study, the desorption efficiency of MDF fines was evaluated using 0.1 M HCl as a desorption agent. Metal ions tend to desorb under acidic conditions due to the competition between the Pb(II) ions and H^+^ ions at the adsorption sites on MDF fines [71].

**Figure 13:**
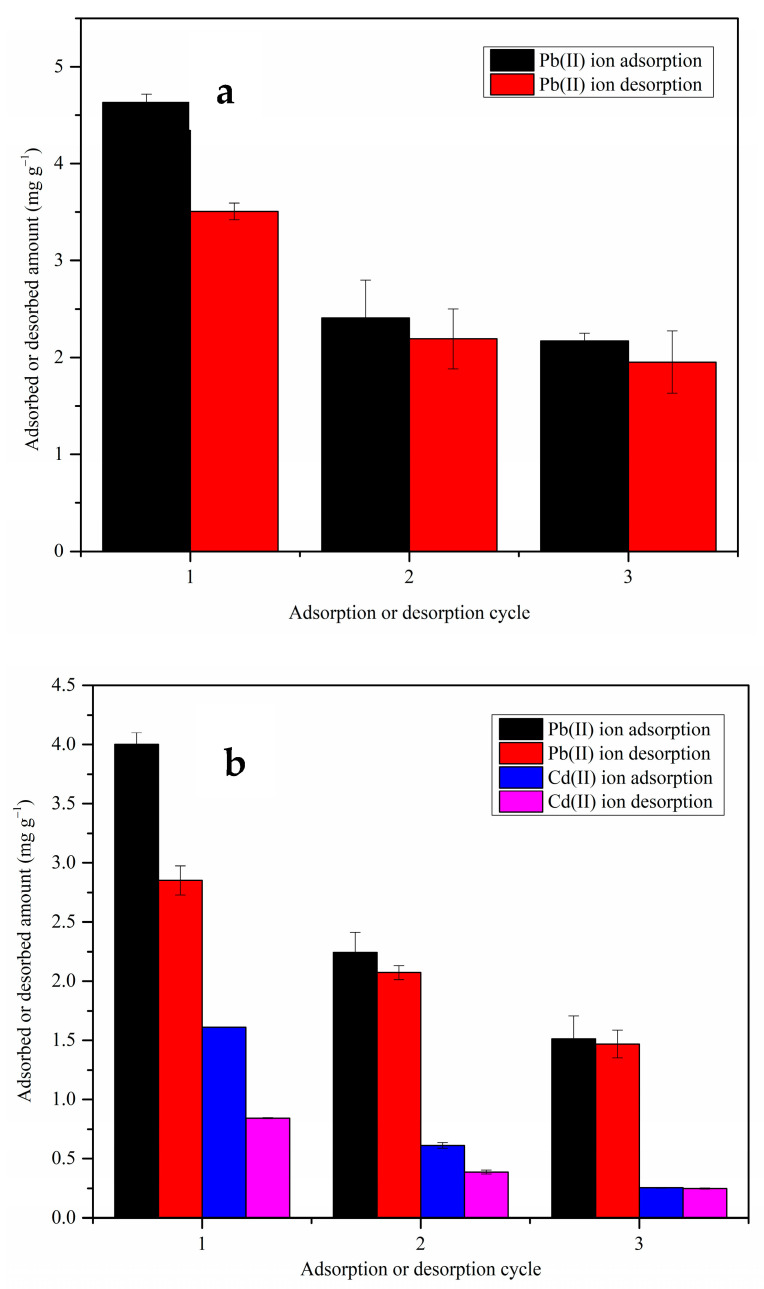
Adsorption/desorption cycles of MDF fines for (**a**) Pb(II) ion adsorption in single-metal ion system; (**b**) Pb(II) and Cd(II) ion adsorption in multi-metal ion system.

**Figure 14:**
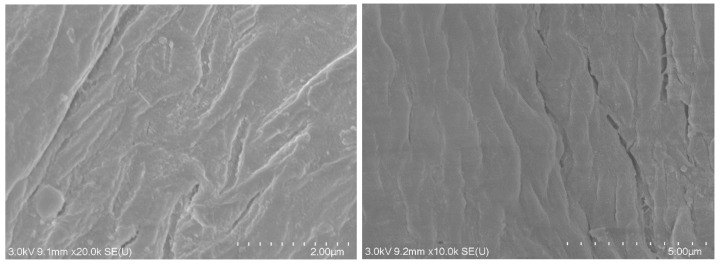
SEM micrographs of MDF fines after the regeneration process.

Significant desorption efficiency was observed for both the single- and multi-metal ion systems (Figure 13). For instance, in the first regeneration cycle of the single-metal ion system (Figure 13a), 76 ± 1.9% of the adsorbed metal ions were desorbed from the adsorbent surface. However, the adsorption efficiency significantly decreased in the subsequent regeneration cycles. The Pb(II) ion adsorption capacity dropped from 4.6 ± 0.1 mg g^−1^ in the first regeneration cycle to 2.4 ± 0.4 and 2.2 ± 0.1 mg g^−1^ in the second and third regeneration cycles, respectively, in the single-metal ion solution systems. This decrease in capability was likely due to the regeneration process deforming the adsorbent surface (Figure 14). Even though the adsorption potential is expected to increase due to the deformations increasing the porosity of the material, it should be noted that the destruction of the adsorbent structure can negatively impact the interactions of the metal ions with the functional groups. The functional groups on the adsorbent surface can be protonated during the desorption process, which makes it difficult for metal ions to be adsorbed onto the surface in the next adsorption cycle. These factors may have caused the decreases in the adsorption capacity in the second and third adsorption cycles. Similar observations have been reported in previous studies [72,73].

The desorption efficiency increased in the second and third regeneration cycles compared to the first regeneration cycle. The % desorption of Cd(II) ions in the multi-metal ion system were 52 ± 0.4%, 63 ± 1.5%, and 97 ± 1.1% in the first, second, and third regeneration cycles, respectively. The literature reports that most of the non-desorbed metal ions are chemically adsorbed and that the physiosorbed metal ions can be easily desorbed from the adsorbent surface [32]. It is suggested that, in the second and third adsorption cycles, metal ions are mainly physically adsorbed onto the MDF surface, providing a high % of desorption during the regeneration process.

Based on the results, HCl can be considered as a potential regeneration agent for MDF fines. The main drawback of this regeneration agent is the reduction in adsorption potential in the subsequent adsorption cycles compared to the first cycle.

#### 3.2.8. Practical Implications and Future Research Directions

The results of this study indicated that MDF fines have the potential for adsorptive removal of Pb(II) ions in both single- and multi-metal (Pb(II)-Cd(II) ions) systems. This is an economical adsorbent for practical applications, as it only requires mechanical processing. The adsorbent must be characterized prior to practical applications to analyze the composition. Based on the results, in practical applications, this material can be used as an initial treatment matrix for heavy metal ion removal to reduce the heavy metal ion load in wastewater. The TOC of the treated water is required to be monitored. Then, treated water can be passed through a more refined adsorbent to complete the treatment process. Selecting an adsorbent that can reduce both heavy metal ion concentrations and TOC levels can be beneficial for the treatment process. The adsorbent can be regenerated using 0.1 M HCl, and regenerated concentrated solution can be used for another application. After the regeneration process, a reduction in adsorption capacity should be expected in practical applications. However, further research (column adsorption analysis, pilot-scale experiments) is required in order to evaluate the suitability of MDF fines for practical applications. Also, further research can be carried out to identify a more efficient regeneration agent for MDF fines.

## 4. Conclusions

In this study, the metal ion adsorption performance and mechanisms of MDF fines were investigated. The adsorbent showed higher adsorption capacity towards Pb(II) ions compared to the other studied metal ions (Cd(II), Cu(II), and Zn(II) ions). Complexation and electrostatic interactions can be suggested as adsorption mechanisms based on the characterization and adsorption analyses. The pseudo-second-order kinetic model and Freundlich isotherm model fit well with the experimental data. The adsorption capacity towards Pb(II) ions was reduced when a competitive metal ion was present in the adsorption system. However, the preferential adsorption of Pb(II) ions was observed in multi-metal ion systems as well. The MDF fines can be regenerated using HCl as a regeneration agent, but a reduction in adsorption potential was observed in the subsequent adsorption cycles. Based on the results, MDF fines can be used as an initial heavy metal ion treatment matrix in wastewater treatment systems to reduce the heavy metal ion load. It is important to monitor the exposure time of the adsorbent to wastewater, as MDF fines can increase the TOC levels in treated water. Then, more refined adsorbents can be used to complete the heavy metal ion treatment process. Further research, such as column adsorption analysis, is required in order to evaluate the suitability of this adsorbent for practical applications.

## Figures and Tables

**Table 1:** FTIR band assignment of MDF fines [40,41].

Band Assignment	Wavenumber (cm^−1^)
O-H stretching	3432
C-H stretching	2918
Aromatic skeletal vibrations	1648, 1512
CH_2_ scissoring and aromatic skeletal vibrations	1459
Aromatic skeletal vibrations	1427
C-H bending	1383
C-O stretching	1263, 1160, 1113, 1059, 1033
C-H bending	897, 617

**Table 2:** Physiochemical properties of the studied metal ions [57].

Physiochemical Property	Metal Ion			
	Cd(II)	Cu(II)	Pb(II)	Zn(II)
Electronegativity (Pauling)	1.69	1.90	2.33	1.65
Hydrated ionic radius (A°)	4.26	4.19	4.01	4.30

**Table 3:** Adsorption kinetic parameters for Pb(II) and Cd(II) ion adsorption on MDF fines.

Metal Ion System	Models	Parameters		R^2^	Error Analysis	
					SSE	HYBRID
Pb single-metal ion system	Pseudo-first-order	*k*_1_	0.34	0.68	0.166	0.956
		*q_e_*	4.24			
	Pseudo-second-order	*k*_2_	0.15	**0.97**	**0.014**	**0.084**
		*q_e_*	4.45			
	Elovich	*α*	1.76 × 10^5^	0.79	0.113	0.724
		*β*	4.04			
Cd single-metal ion system	Pseudo-first-order	*k*_1_	0.65	0.76	0.002	0.021
		*q_e_*	2.83			
	Pseudo-second-order	*k*_2_	1.47	**0.99**	**0.0002**	**0.003**
		*q_e_*	2.84			
	Elovich	*α*	3.05 × 10^34^	0.85	0.001	0.013
		*β*	30.82			
Pb multi-metal ion system	Pseudo-first-order	*k*_1_	0.39	0.94	0.055	0.361
		*q_e_*	3.92			
	Pseudo-second-order	*k*_2_	0.26	**0.99**	**0.005**	**0.030**
		*q_e_*	4.01			
	Elovich	*α*	1.96 × 10^6^	0.89	0.056	0.358
		*β*	5.21			
Cd multi-metal ion system	Pseudo-first-order	*k*_1_	0.33	0.48	0.023	0.357
		*q_e_*	1.58			
	Pseudo-second-order	*k*_2_	0.52	**0.98**	**0.002**	**0.025**
		*q_e_*	1.65			
	Elovich	*α*	1.75 × 10^7^	0.78	0.016	0.293
		*β*	14.46			

Note: The bold values represent the R^2^ values and SSE and HYBRID error values of the best fit kinetic model.

**Table 4:** Adsorption isothermal parameters for Pb(II) ion adsorption on MDF fines.

Models	Parameters		R^2^	Error Analysis	
				SSE	HYBRID
Langmuir	*q_m_*	7.973	0.86	5.94	22.11
	*b*	0.429			
Freundlich	*K*	3.708	**0.99**	**0.37**	**1.32**
	*n*	5.442			
Temkin	*B*	2.196	0.98	1.28	4.63
	*K_T_*	19.415			

Note: The bold values represent the R^2^ value and SSE and HYBRID error values of the best fit isotherm model.

## Data Availability

Data are contained within the article.
